# What are the characteristics of an application (or team) which are associated with success or failure of an application to the NIHR HTA programme for research funding?

**DOI:** 10.1186/1745-6215-12-S1-A58

**Published:** 2011-12-13

**Authors:** Sheila Turner, Peter Davidson, Louise Dent, Elke Streit, Victoria Bowness

**Affiliations:** 1 NIHR Evaluation, Trials and Studies Coordinating Centre at the University of Southampton SO16 7NS, UK; 2 University of Southampton Clinical Trials Unit, Southampton, SO16 6YD, UK

## Background

Previous studies have shown that gender or seniority of applicants can be linked to success in obtaining research funding, but we found little evidence about other features of successful applications for trials funding. The aim of this study was to investigate the association between items or features in a funding application and success when considered by a research funding board. This knowledge will be useful to potential applicants to the HTA programme and to other programmes, and to funding boards of this and other research programmes. The work was not intended to set ideal standards for applications, rather to extend knowledge of current practice and to explore what may be learnt from this.

## Methods

We undertook a retrospective cohort study using applications to the NIHR HTA programme. The sample comprised 296 outline proposals for primary research, submitted to the commissioning board of the HTA programme between January 2006 and December 2009. Proposals to the commissioned work stream were selected as these proposals addressed issues which the HTA programme already deemed to be important, hence the priority of the research question was not considered as one of the selection criteria for success or failure.

The dependent variables were characteristics of the applications (or teams) selected a priori by a panel of experts drawn from the board and informed by existing literature. They included characteristics relating to the principal investigator, the team and the bid. The outcomes were success or failure at short-listing and in obtaining research funding.

Data were analysed using uni-variate and multivariate analysis and conditional logistic regression modelling.

## Results

Results of the analysis indicated that the characteristics most strongly associated with success were: whether the proposal met the specifications of the commissioning brief; multi-disciplinarity of the team; experience of writing and critiquing research bids; and whether or not a pilot or feasibility study had been conducted. The gender of the chief investigator and proposed trial costs were not significantly associated with success or failure.

## Conclusions

The association between meeting the commissioning brief and success is in line with expectations and is reassuring regarding the decision making processes of the board. It suggests to applicants that deviating from the brief is risky. The relative success of multi-disciplinary teams and those including a statistician, a health economist or another methodologist were also anticipated by the experts. This offers evidence to applicants of the preferred make-up of teams. Further research in other funding streams or organisations would be useful.

